# PRPF19 modulates morphology and growth behavior in a cell culture model of human skin

**DOI:** 10.3389/fragi.2023.1154005

**Published:** 2023-05-05

**Authors:** Lisa Kleissl, Regina Weinmüllner, Ingo Lämmermann, Ruth Dingelmaier-Hovorka, Mohammad Jafarmadar, Abdoelwaheb El Ghalbzouri, Georg Stary, Johannes Grillari, Hanna Dellago

**Affiliations:** ^1^ Ludwig Boltzmann Institute for Rare and Undiagnosed Diseases, Vienna, Austria; ^2^ Department of Dermatology, Medical University of Vienna, Vienna, Austria; ^3^ CeMM Research Center for Molecular Medicine of the Austrian Academy of Sciences, Vienna, Austria; ^4^ Institute of Molecular Biotechnology, University of Natural Resources and Life Sciences, Vienna, Austria; ^5^ Christian Doppler Laboratory for Biotechnology of Skin Aging, Vienna, Austria; ^6^ Ludwig Boltzmann Institute for Traumatology in cooperation with AUVA, Vienna, Austria; ^7^ Department of Dermatology, Leiden University Medical Center, Leiden, Netherlands

**Keywords:** PRPF19, SNEV, DNA damage repair, aging, wound healing, fibroblast, DNA repair factor

## Abstract

The skin provides one of the most visual aging transformations in humans, and premature aging as a consequence of oxidative stress and DNA damage is a frequently seen effect. Cells of the human skin are continuously exposed to endogenous and exogenous DNA damaging factors, which can cause DNA damage in all phases of the cell cycle. Increased levels of DNA damage and/or defective DNA repair can, therefore, accelerate the aging process and/or lead to age-related diseases like cancer. It is not yet clear if enhanced activity of DNA repair factors could increase the life or health span of human skin cells. In previous studies, we identified and characterized the human senescence evasion factor (SNEV)/pre-mRNA-processing factor (PRPF) 19 as a multitalented protein involved in mRNA splicing, DNA repair pathways and lifespan regulation. Here, we show that overexpression of PRPF19 in human dermal fibroblasts leads to a morphological change, reminiscent of juvenile, papillary fibroblasts, despite simultaneous expression of senescence markers. Moreover, conditioned media of this subpopulation showed a positive effect on keratinocyte repopulation of wounded areas. Taken together, these findings indicate that PRPF19 promotes cell viability and slows down the aging process in human skin.

## 1 Introduction

The human skin is a huge organ composed of an epidermal and a dermal layer that blend into each other and the subcutis located below. Its thickness varies significantly depending on the location in the body. The main functions of the skin are homeostasis of body temperature, oxygen uptake, pressure sensation, and barrier function. To guarantee all these features, it is important that the hierarchical structure of the skin is maintained (Ulfig, 2005).

Fibroblasts are irregular spindle-shaped cells that express mesenchymal markers like vimentin and collagen I and exhibit radial growth (Sriram et al., 2015). They are the main resident cell types in the connective tissue of the human dermis, contributing to extracellular matrix (ECM) production, hair follicle initiation, and cycling (Zou et al., 2021). While for a long time considered a simple static cell population, more recently, skin fibroblasts have been shown to be heterogeneous cells with subpopulations located in the superficial papillary (papillary fibroblast) and the deep reticular (reticular fibroblast) dermis (Sorrell and Caplan, 2004a; Thulabandu et al., 2018), with distinct characteristics (Sorrell et al., 2004). Papillary fibroblasts are thin, spindle-shaped cells, exhibiting an increased proliferation and decreased contraction capacity compared to reticular fibroblasts *in vitro* (Harper and Grove, 1979; Azzarone et al., 1982). When cultivated in monolayer cultures, they reach higher cell density (Sorrell et al., 2004). Reticular fibroblasts are more flattened and widespread and typically express more *a*-smooth muscle actin (α-SMA), a marker for myofibroblasts (Janson et al., 2012), and also induce faster contraction of type I collagen lattices (Sorrell et al., 1999). In accordance with these observations, reticular fibroblasts overexpress genes involved in cell motility, whereas papillary fibroblasts show increased expression of genes involved in immune response (Janson et al., 2012). Alongside their distinct ECM synthesis, the two subpopulations exhibit different patterns of cytokine and growth factor secretion important for the crosstalk with neighboring cells (Sorrell et al., 2004). Fibroblasts influence keratinocyte behavior by secretion of growth factors like keratinocyte growth factor (KGF) and transforming growth factor (TGF) *ß*. As fibroblast subpopulations secrete growth factors in various amounts, it appears that they influence epidermal proliferation and differentiation to a variable extent, and a finely tuned balance between pro- and anti-proliferative signals is necessary to maintain epidermal homeostasis (Sorrell et al., 2004).

Human skin cells are continuously exposed to exogenous and endogenous DNA-damaging agents, such as ultraviolet radiation (UV) as well as reactive oxygen species (ROS), which can cause DNA damage in all phases of the cell cycle (Kakarougkas and Jeggo, 2014). Failure of the DNA damage response (DDR) promotes pathophysiological outcomes such as cancer, hereditary disorders like Werner’s or Bloom’s syndrome (Bhattacharjee and Nandi, 2018), and premature aging (Garinis et al., 2008; Dellago et al., 2012; Mahajan, 2015). However, if the mammalian cell response works properly after DNA damage, the DNA damage checkpoint pathways initiate signaling cascades resulting in cell cycle arrest as well as recruitment of damage-specific repair complexes (Kakarougkas and Jeggo, 2014), which act together to catalyze the restoration of genomic integrity (Motoyama and Naka, 2004; Howard et al., 2015). During aging, the human skin undergoes several changes like progressive thickness decrease and loss of epidermal ridges (Lavker et al., 1987; Janson et al., 2013a). This phenomenon is caused by changes in the cellular composition and proteoglycans of the human dermis during aging (Sorrell and Caplan, 2004a; Janson et al., 2013a; Driskell et al., 2013).

ScPSO4 was first identified in *Saccharomyces cerevisiae* as psoralen-sensitive mutant 4. As a mutant strain, it was unable to repair photoactivated 8 methoxy psoralen-induced DNA interstrand crosslinks (ICL), and the gene was found to be allelic to prp19 in haploid cells and essential for mRNA splicing (da Silva et al., 1995; Grey et al., 1996). Its enzymatic activity is similar to that of an E3 ubiquitin ligase carrying a U-box domain (de Moura et al., 2018; Hatakeyama and Nakayama, 2003b). The human ortholog PRPF19 has been similarly shown to be a multi-talented protein localized mainly to the nucleus and, therefore, also termed nuclear matrix protein (hNMP200) (Gotzmann et al., 2000). It was also identified as a DNA repair factor termed hPso4 (Mahajan and Mitchell, 2003), a protein extending the replicative lifespan of human endothelial cells and termed senescence evasion factor (SNEV) (Grillari et al., 2005), and an essential mRNA splicing factor (Prp19) (Chan et al., 2003) whose knock-out is early embryonic lethal (Fortschegger et al., 2007). Splicing is probably its best characterized function and is a critical process for genome stability and reported to also play a role in aging and certain diseases (Chanarat and Sträßer, 2013; Angarola and Anczuków, 2021). Its role in DNA damage repair is connected to its interaction with terminal deoxynucleotidyl transferase (TdT) to repair double-strand breaks (Mahajan and Mitchell, 2003) or acts as a complex with other PPRF19-associated proteins to repair DNA interstrand cross-links (Zhang et al., 2005). These functions ultimately might have an impact on aging and lifespan (Grillari et al., 2000; Voglauer et al., 2006a) and have recently been reviewed in Idrissou and Maréchal (2022). It is known that mutations and deletions of various DNA repair factors cause progeroid syndrome and premature aging. We have previously shown that overexpression of the DNA repair factor PRPF19 has a lifespan-prolonging effect on human umbilical vein endothelial cells (Voglauer et al., 2006a). Here, we explore the role of PRPF19 in skin aging, using a dermal fibroblast cell culture model.

## 2 Materials and methods

### 2.1 Cell isolation and cell culture conditions

Human dermal fibroblasts (HDF)76 were isolated from a human skin biopsy sample of a 58-year-old healthy female donor. HDF161 were isolated from a human skin biopsy sample of a 65-year-old healthy female donor. HDFs were obtained from Evercyte (Vienna, Austria). Site-matched papillary and reticular HDF were isolated from the dermis as described (Janson et al., 2012), and their identity was confirmed by measuring the expression levels of three papillary and three reticular mRNA markers (Lämmermann et al., 2018). The site-matched papillary and reticular HDFs were isolated from surplus tissue of healthy donors by the Department of Dermatology of the Leiden University Medical Center (Leiden, Netherlands) according to Article 467 of the Dutch Law on Medical Treatment Agreement and the Code for Proper Use of Human Tissue of the Dutch Federation of Biomedical Scientific Societies. All fibroblast strains were cultivated in Dulbecco’s modified Eagle’s medium (DMEM) and Ham’s F12 (1:1) (Merck Millipore, Germany), supplemented with 10% fetal calf serum (FCS) (Sigma-Aldrich, Missouri, United States) and 4 mM l-glutamine (Sigma-Aldrich, Missouri, United States).

Normal human epidermal keratinocytes (NHEKs) were isolated from human skin tissue samples. The cells had previously been transfected with a plasmid encoding the SV40 Early Region, followed by transfection with human telomerase reverse transcriptase (hTERT) to generate an immortal cell line. These cells were cultivated in keratinocyte growth media 2 (KGM-2) (Lonza, Switzerland). All cells were tested for mycoplasma at regular intervals. Isolation of the cells was approved by the respective local ethics commission, and all donors gave informed consent. Thus, this study was performed in compliance with the Declaration of Helsinki.

Confluent adherent cultures were detached using 0.1% trypsin and 0.02% EDTA (Sigma-Aldrich, United States) in PBS for 5 min at 37°C and were passaged in an appropriate split ratio reaching from 1:2 to 1:5 approximately twice a week depending on confluence and population doubling (PD) level.

To induce apoptosis, young to middle-aged fibroblasts were seeded into chamber slides (ibidi 15 µL-slide 8-well, Germany). After 24 h, cells were treated with 50 µM cisplatin (Sigma-Aldrich, Missouri, United States) for 3 h to assess apoptosis induction by DNA double-strand breaks.

### 2.2 Plasmid construction, generation of recombinant retroviruses, and cell line establishment

For fibroblast cell type establishment, HDF76 at PD10.5 and HDF161 at PD9 were infected with lentiviral particles containing one of three different genetic constructs, empty vector, wild-type PRPF19, or a mutated form of PRPF19 that cannot be phosphorylated by ATM. Therefore, the serine at position 149 was mutated to alanine using the QuickChange Multi Site-Directed Mutagenesis Kit (Agilent, Santa Clara, United States). PRPF19 cDNA was amplified and ligated into the retroviral plasmid pLenti6. Described particles were provided by the cooperating laboratory of Jansen-Duerr (University of Innsbruck). For the establishment of the three cell strains, 40 µL of lentivirus particles (MOI 4) were mixed with 1 ml culture media supplemented with 6 μg/ml polybrene, and the mixture was added to HDF in three parallel approaches for the three fibroblast types. Thereafter, transfectants were selected using 5 μg/ml Blasticidin (InvivoGen, California, United States). Arising cell clones, transfected with either of the constructs, were grown as mass cultures, and PDs post transfection (PDpT) were calculated starting with the first passage after selection was completed. Transduced HDF76 fibroblasts were used for all experiments if not stated otherwise.

### 2.3 Antibodies

SNEV/Prp19/Pso4 rabbit polyclonal antibody was obtained from Bethyl Laboratories (Montgomery, TX, United States) #A300-102A. Gamma H2A.X (phosphor S139) mouse monoclonal antibody $\left[9F3\right]$ was obtained from Abcam (Cambridge, UK) #26350. Alexa Fluor^®^ 488 mouse anti-human vimentin antibody was obtained from BD Pharmingen™ (BD Biosciences, California, United States) #562338. Actin monoclonal antibody, *a*-smooth muscle clone 1A4, was obtained from Sigma-Aldrich (Merck, Germany) #A2547. Alexa Fluor^®^ 488 donkey anti-rabbit was obtained from Jackson ImmunoResearch (West Grove, PA, United States) #711–545–152.

### 2.4 Indirect immunofluorescence staining

Cells were seeded into chamber slides (ibidi 15 µL-slide 8-well, Germany) and cultivated 24–48 h so that single cells are still easily discernible. Next, cells were washed twice with PBS and fixed in 4% paraformaldehyde (ROTI^®^ Histofix, Carl Roth, Germany) for 10 min at room temperature. Permeabilization was performed with 0.3% Triton-X-100 in PBS +10% FCS for 10 min at room temperature. Cells were incubated for 1 h at 37°C, with the first antibody diluted in 0.3% Triton-X-100 + 10% FCS. With subsequent washing, if necessary, cells were incubated with the appropriate secondary antibody diluted in PBS + 10% FCS for another hour at 37°C. Alexa Fluor^®^ anti-vimentin was diluted 1:250, anti-α-smooth muscle actin was diluted 1:250, its respective secondary antibody Alexa Fluor^®^ 488 donkey anti-mouse 1:500, SNEV/Prp19/Pso4 was diluted 1:100, and its respective secondary antibody Alexa Fluor^®^ 488 donkey anti-rabbit 1:500. To visualize the nuclei, cells were counterstained with 100 ng/ml 4′,6-diamidino-2-phenylindole (DAPI) in PBS for 10 min at room temperature. After staining, slides were mounted on coverslips using SlowFade™ Gold Antifade Mountant (Life Technologies, Grand Island, NY, United States). Microscopy and image analysis were carried out using a Leica SP5 II laser scanning confocal microscope (Leica Microsystems CMS, Mannheim, Germany). The cell image analysis software CellProfiler was used to quantify parameters reflecting fibroblast morphology. Once the correct settings are entered into the software, it automatically recognizes the area of each cell and calculates the respective parameters reflecting fibroblast morphology. In this case, compactness was defined as the variance of the radial distribution normalized by the area, meaning the higher the value, the more elongated the cell. The eccentricity is the ratio of the distance between the foci of the ellipse and its major axis length. The value can range from 0 to 1, meaning the lower the value, the more circular the cell. To quantify the fluorescence intensity, ImageJ was used. Each cell area was individually marked, and the fluorescence intensity was measured.

### 2.5 Proliferation assay

Cells were seeded in equal numbers into six-well plates, and cell numbers per milliliter were determined after 6, 10, and 16 days. A live–dead cell staining and quantification by trypan blue dye exclusion method was performed using the Vi-CELL-XR cell viability analyzer (Beckman Coulter Life Sciences, United States).

### 2.6 Quantitative real-time PCR

Cells were lysed in TRI Reagent (Sigma-Aldrich, Missouri, United States), and RNA was isolated following the manufacturer’s protocol. RNA quality and concentration were measured with a NanoDrop One UV–Vis Spectrophotometer (Thermo Scientific, Massachusetts, United States). cDNA was synthesized from 500 ng of total RNA with the High-Capacity cDNA Reverse Transcription Kit (Applied Biosystems, California, United States). Quantification was conducted with the 5x HOT FIREPol^®^ EvaGreen^®^ qPCR Mix Plus with ROX (Solis BioDyne, Estonia) using the Rotor-Gene Q (QIAGEN, Netherlands) and the respective primer pairs (Table 1). Expression values were normalized to GAPDH mRNA.

**TABLE 1:** Sequences of RT-qPCR primers.

Gene name	Sense primer	Anti-sense primer
GAPDH	CGA​CCA​CTT​TGT​CAA​GCT​CA	TGT​GAG​GAG​GGG​AGA​TTC​AG
PDPN	GCA​TCG​AGG​ATC​TGC​CAA​CT	CCC​TTC​AGC​TCT​TTA​GGG​CG
NTN1	TGC​CAT​TAC​TGC​AAG​GAG​GG	TTG​CAG​GTG​ATA​CCC​GTC​AC
TGM2	GGC​GAA​CCA​CCT​GAA​CAA​AC	AGG​ATG​CAA​AGA​GGA​ACG​CT
PPP1R14A	GTG​GAG​AAG​TGG​ATC​GAC​GG	CCC​TGG​ATT​TTC​CGG​CTT​CT
p53	GCTTTCCACGACGGTGAC	GCT​CGA​CGC​TAG​GAT​CTG​AC
p21	GGC​GGC​AGA​CCA​GCA​TGA​CAG​ATT	GCA​GGG​GGC​GGC​CAG​GGT​AT
CXCL8	CTC​TTG​GCA​GCC​TTC​CTG​ATT​T	ACA​GAG​CTC​TCT​TCC​ATC​AGA
COMP	CCC​AGA​AGA​ACG​ACG​ACC​AA	AGT​CCT​GAT​GTC​CGT​CTC​CA
MMP3	GGT​TCC​GCC​TGT​CTC​AAG​AT	AGG​GAT​TTG​CGC​CAA​AAG​TG
MMP1	CCA​GGT​ATT​GGA​GGG​GAT​GC	CGA​TGG​GCT​GGA​CAG​GAT​TT
PRPF19 (SNEV)	TCA​TTG​CCC​GTC​TCA​CCA​AG	GGC​ACA​GTC​TTC​CCT​CTC​TTC
CXCL1	TCA​ATC​CTG​CAT​CCC​CCA​TAG	CAG​GAA​CAG​CCA​CCA​GTG​AG
CCL2	GAA​AGT​CTC​TGC​CGC​CCT​TC	ACA​GAT​CTC​CTT​GGC​CAC​AA

### 2.7 Apoptosis staining/SA-β-gal staining

To assess the apoptosis level, the SA-β-gal Staining Kit from Invitrogen (California, United States) was used. Cells were washed with PBS and fixed for 10 min in 2% formaldehyde and 0.2% glutaraldehyde in PBS. Cells were washed twice with PBS and once with staining buffer, comprising 100 mM citrate buffer and 200 mM sodium phosphate at pH 6. Then cells were incubated overnight at 37°C with a staining solution composed of 400 mM sodium ferricyanide, 400 mM sodium ferrocyanide, 200 mM magnesium chloride, X-gal, and staining buffer. Pictures were taken from random positions all over the well, and after randomization, positive and negative cells were counted in blinded fashion.

### 2.8 Scratch assay

NEHK/SVTERT were cultivated in 6-well plates until 90%–100% confluence. Thereafter, the supernatant was discarded, and a scratch of around 5 mm in length was made through the cell monolayer. After washing with PBS, cells were cultivated with 2 ml conditioned medium, and pictures were taken for 24 h with a light microscope (Leica DM IL LED, Germany). Conditioned medium was generated by seeding the same number of untransduced control, PRPF19wt, and PRPF19mut human dermal fibroblast cells and cultivating in KGM-2 (Lonza, Switzerland) for 48 h at 37°C. Due to the distinct proliferation capacities of the three different fibroblast strains (see also Figure 2), cell numbers were collected again at the time of media harvest, and conditioned medium of the individual cell strains was normalized to the smallest cell number observed to exclude effects caused due to more/less concentrated condition media. We normalized the condition media, diluting it with KGM-2, treated the same way as the conditioned media (incubation at 37°C for 48 h) to make the results comparable. Conditioned media were harvested from the cells and centrifuged (300 x g 5 min) to get rid of cell debris before directly adding the supernatant conditioned media to the scratched keratinocyte layer. The images were analyzed to determine the scratch area over time using ImageJ. The extent of wound healing was ascertained as a percentage of the initial scratch area.

### 2.9 Full-thickness human skin equivalent

Full-thickness human skin equivalents were generated as published in Mildner et al. (2006). Briefly, 2.5 × 10^5^ fibroblasts were seeded into a collagen gel comprising eight parts bovine collagen I 3 mg/ml (Advanced BioMatrix, Carlsbad, CA, United States), one part 10X HBSS (Thermo Fisher Scientific, Waltham, MA, United States), and one part of FCS (Thermo Fisher Scientific, Waltham, MA, United States). The gel was equilibrated for at least 1 h at 37°C with KGM-2 supplemented with KGM-2 BulletKit (Lonza, Basel, CH), followed by the seeding of 1.5 × 10^6^ keratinocytes on the top of each dermal equivalent. On day 3, the skin equivalents were lifted to the air–liquid interface, and the medium was changed to KGM supplemented with KGM BulletKit (Lonza, Basel, CH), except for bovine pituitary extract. In addition, 50 μg/ml L-ascorbic acid (Sigma-Aldrich, St. Louis, MO, United States), 0.1% bovine serum albumin (Sigma-Aldrich, St. Louis, MO, United States), 10 μg/ml transferrin (Lonza, Basel, CH), and 1.3 mM calcium chloride (Lonza, Basel, CH) were supplemented to the media to facilitate epidermal differentiation. Skin equivalents were cultured for 10 days on cell culture inserts (Falcon/Corning, NY, United States) in deep well plates (Falcon/Corning, NY, United States), and the medium was refreshed every other day. After 10 days, the skin equivalents were harvested, formalin-fixed, and paraffin-embedded for hematoxylin and eosin staining and further histological analysis. The average epidermal thickness was calculated by the height of the originated frame of the epidermis using ImageJ.

### 2.10 TGF-β ELISA

Conditioned media was generated, as mentioned previously. KGM-2 media alone was treated the same way, serving as the negative control. To determine the concentration of activated human TGF-β1, the Quantikine™ ELISA Human TGF-β1 Immunoassay was used (R&D Systems, Minneapolis, MIN, United States) according to the manufacturer’s protocol. Briefly, first the sample was activated with 1 N HCl and then neutralized with 1.2 N NaOH/0.5 M HEPES before adding them together with the standard dilutions to the microplate. Optical density of the samples was measured within 30 min in the POLARstar^®^ Omega plate reader (BMG Labtech, Ortenberg, GER). Subsequently, the standard curve was created, and TGF-β concentrations were calculated.

### 2.11 Statistical Analysis

Statistical analyses were performed using GraphPad Prism software. Error bars represent the standard error of the mean (± SEM) or standard deviation (+SD) calculated using Prism. Statistical tests used in this study were paired Student’s t-test, one-way and two-way ANOVA with multiple comparisons, a mixed model with multiple comparisons, and a Chi-squared test unless stated otherwise. *p*-values < 0.05 were considered statistically significant.

## 3 Results

### 3.1 Generation and verification of PRPF19 overexpression in human dermal fibroblasts

**FIGURE 1:**
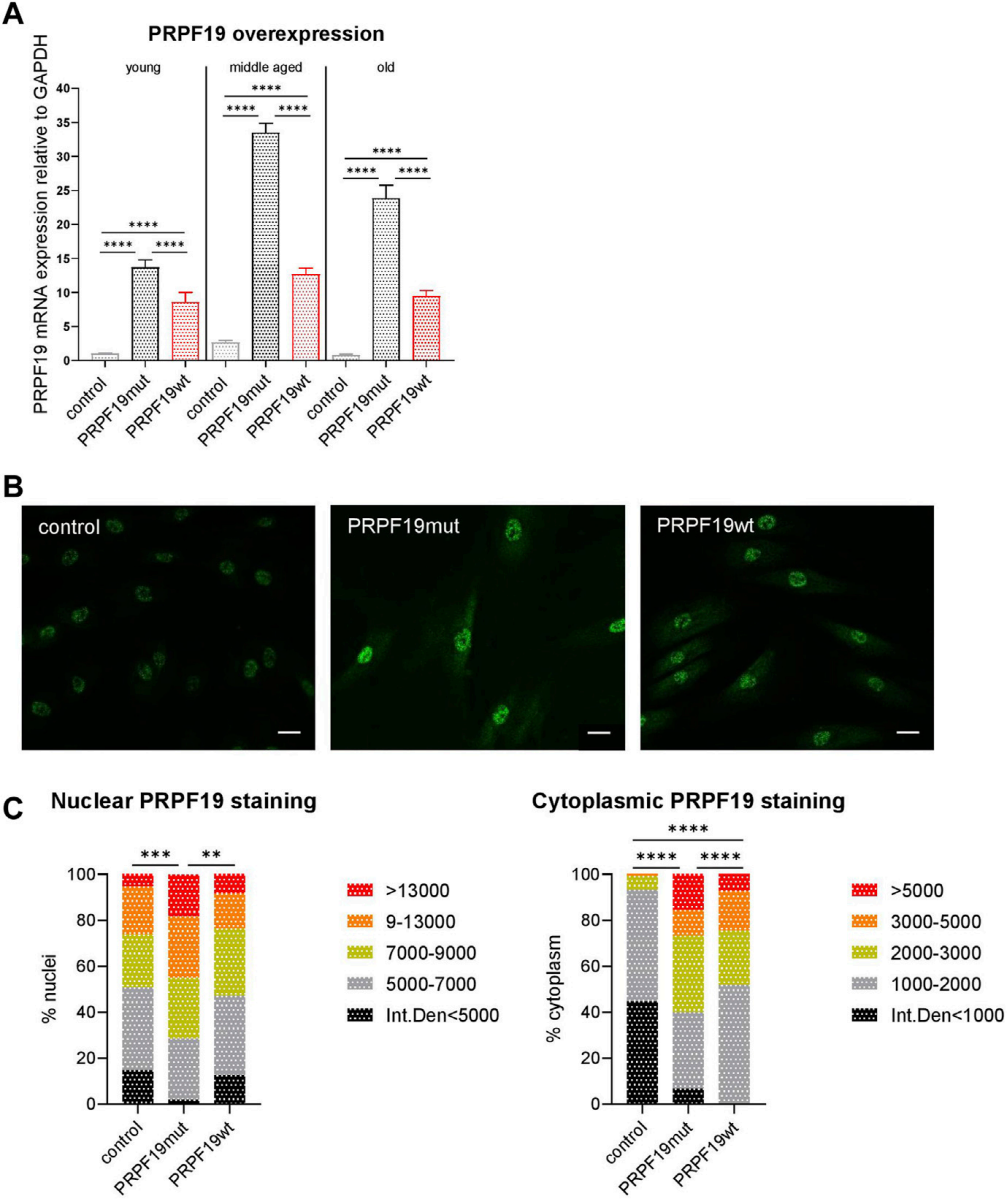
Generation and verification of PRPF19 overexpression in human dermal fibroblasts. Human dermal fibroblasts from the same donor were transduced with one of three different lentiviral constructs. **(A)** PRPF19 mRNA expression of the three generated fibroblast strains at low, middle, and high PDpT. All data are plotted as means + SD. One-way ANOVA with Tukey correction for multiple comparisons. Data are presented as expression level normalized to young control. **(B)** Representative immunofluorescence images of nuclear and cytoplasmic PRPF19 and **(C)** quantification of nuclear and cytoplasmic pixel intensity. Statistical significance was determined using the chi square test for the comparison of pixel intensity. n = 2, with six to eight pictures analyzed for each replicate. All the data are expressed as means; **p < 0.01; ***p < 0.001; ****p < 0.0001. Scale bar 100 µm.

**FIGURE 2:**
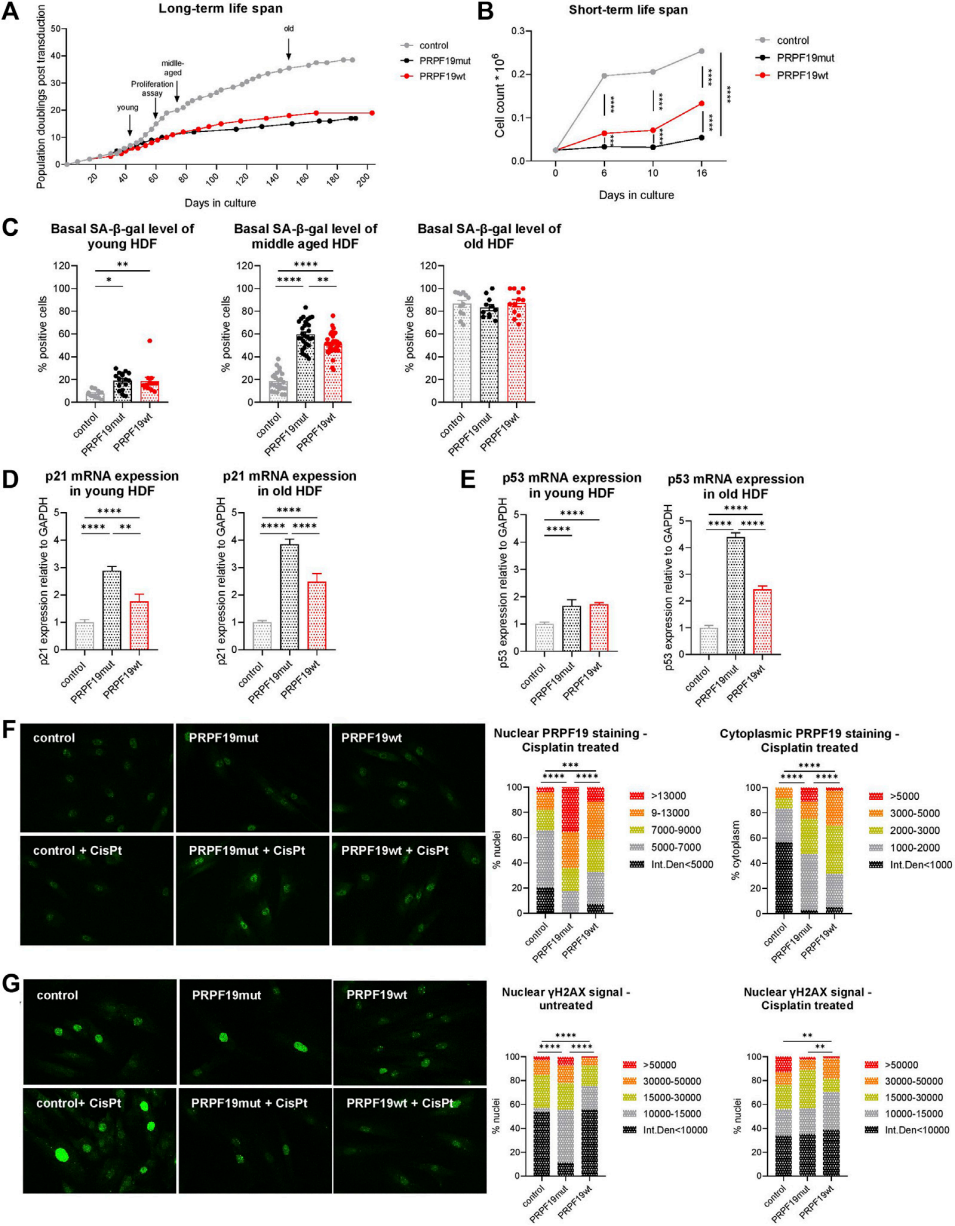
PRPF19 stable overexpression reduces the replicative lifespan of human dermal fibroblasts but protects from DNA damage. **(A)** Long-term growth curve over 5 months calculated on the basis of PDpT and **(B)** proliferation assay of the three generated fibroblast strains at three time points over 16 days. Arrows indicate timeframes at which fibroblasts are considered young, middle-aged, and old and the time point for the proliferation assay. For **(B)** all data are plotted as means ± SEM. Statistical significance was determined by two-way ANOVA with Tukey correction for multiple comparisons (n = 3). **(C)** SA-β-gal staining, a senescent marker, on young, middle-aged, and old transduced fibroblast strains throughout their cellular lifespan. n = 3, with an average of 10 pictures analyzed for each replicate. Data are plotted as individual values ±SEM. Statistical significance was determined by fitting a mixed model with Tukey correction for multiple comparisons. mRNA expression of **(D)** p21 and **(E)** p53 in young and old fibroblast strains. Data are presented as expression level normalized to control and are plotted as means +SD. Statistical significance was determined by one-way ANOVA with Tukey correction for multiple comparisons. Representative immunofluorescence images and quantification of nuclear and cytoplasmic **(F)** PRPF19 and **(G)** γH_2_AX staining before and after cisplatin treatment for 24 h (see also Supplementary Figure S1). Statistical significance was determined using the chi square test for the comparison of pixel intensity. n = 2 with five to eight pictures analyzed for each replicate. Data are expressed as means; *p < 0.05; **p < 0.01; ***p < 0.001; ****p < 0.0001. Microscopic pictures were taken at ×100 magnification.

To study the role of PRPF19 in skin cells, stable fibroblast types overexpressing PRPF19 were established. Human dermal fibroblast strains (HDFs) of healthy donors characterized earlier (Narzt et al., 2021) were transduced with lentiviral particles carrying one of three different constructs, either an empty control vector (referred to as “control” hereafter), wild-type PRPF19 (PRPF19wt), or a mutant form of PRPF19 that cannot be phosphorylated at serine 149A (S149A) by ataxia telangiectasia mutated (ATM) (Dellago et al., 2012) (PRPF19mut). The effect of phosphorylation-incompetent PRPF19 was analyzed as it was known from previous experiments that the phosphorylation of PRPF19 at the ATM target site is important for growth regulation and stress repair (Dellago et al., 2012). Furthermore, the PRPF19mut served as a non-functional mutant and helped dissect functions dependent on ATM-mediated phosphorylation. PRPF19 overexpression was verified by reverse transcription–quantitative PCR (RT-qPCR) throughout the replicative lifespan of human dermal fibroblasts (Figure 1A). Young, middle-aged, and old cells refer to fibroblasts generated from the same donor with low, middle, and high population doublings post-transduction (PDpT) (see also Figure 2A). In addition, we complemented this analysis with immunofluorescence staining for PRPF19, confirming PRPF19 overexpression on protein level by increased signal intensities in the nuclei and the cytoplasm of PRPF19wt and PRPF19mut cell strains as compared to control (Figures 1B, C).

Taken together, we confirmed the stable overexpression of wild-type PRPF19 and phosphorylation insufficient PRPF19 in human dermal fibroblasts using independent methods. The established transduced cell strains were used for all further experiments.

### 3.2 PRPF19 stable overexpression reduces the replicative lifespan of human dermal fibroblasts but protects from DNA damage

In order to test the effect of stable PRPF19 overexpression on cellular lifespan, we cultivated the recombinant fibroblast cell types for more than 5 months and generated growth curves on the basis of PDpT. In contrast to our previous findings in human endothelial cells (Voglauer et al., 2006b), both PRPF19wt and PRPF19mut cells underwent fewer population doublings than control cells (Figure 2A, Supplementary Figure S1), having a growth curve similar to non-transduced HDF from the same donor observed in previous studies (Narzt et al., 2021; Lämmermann et al., 2018). This was accompanied by a reduced growth rate as assessed by proliferation assays over 16 days (Figure 2B) as well as by higher levels of the senescence marker SA-β-galactosidase (SA-β-gal) in young and middle-aged PRPF19wt and PRPF19mut cells compared to control (Figure 2C), which reached a plateau versus the end of their replicative lifespan. Using qPCR, we demonstrate that both PRPF19wt and PRPF19mut strains were associated with increased levels of cyclin-dependent kinase inhibitor 1 (p21) and the tumor-suppressor gene p53 throughout the cellular lifespan (Figures 2D,E). This is in line with recent findings that ATM is dispensable for p53–p21 pathway activation (Yano et al., 2021). In order to test if the overexpression of PRPF19 would still decrease markers of DNA damage as observed previously with endothelial cells (Voglauer et al., 2006b), we treated the cells for 24 h with cisplatin, a known inducer of DNA double-strand breaks and interstrand cross-links, and checked for PRPF19 expression levels as well as for DNA damage by immunofluorescence staining. Nuclear and cytoplasmic PRPF19 levels were higher in PRPF19wt and PRPF19mut cells than in control, before (as expected by their overexpression) (Figure 1B) and after cisplatin treatment (Figure 2F lower panel, Supplementary Figure S1). Under basal conditions, immunofluorescence staining for gamma-histone H_2_AX (γH_2_AX), which is induced/phosphorylated as a result of DNA double-strand breaks, was reduced in PRPF19wt while surprisingly being higher in PRPF19mut as compared to control (Figure 2G upper image panel, left bar chart). After cisplatin treatment, PRPF19wt, as well as PRPF19mut, are protected from γH_2_AX induction, while this effect was not observed in control (Figure 2G, Supplementary Figure S1).

These data indicate that PRPF19 overexpression promotes DNA damage repair. Moreover, after the induction of DNA double-strand breaks, this process seems to be even independent of its phosphorylation at the ATM target site as we also observed lower levels of DNA double-strand breaks in PRPF19mut after cisplatin treatment (Supplementary Figure S1).

### 3.3 PRPF19 induces a papillary/juvenile morphology in human dermal fibroblasts

**FIGURE 3:**
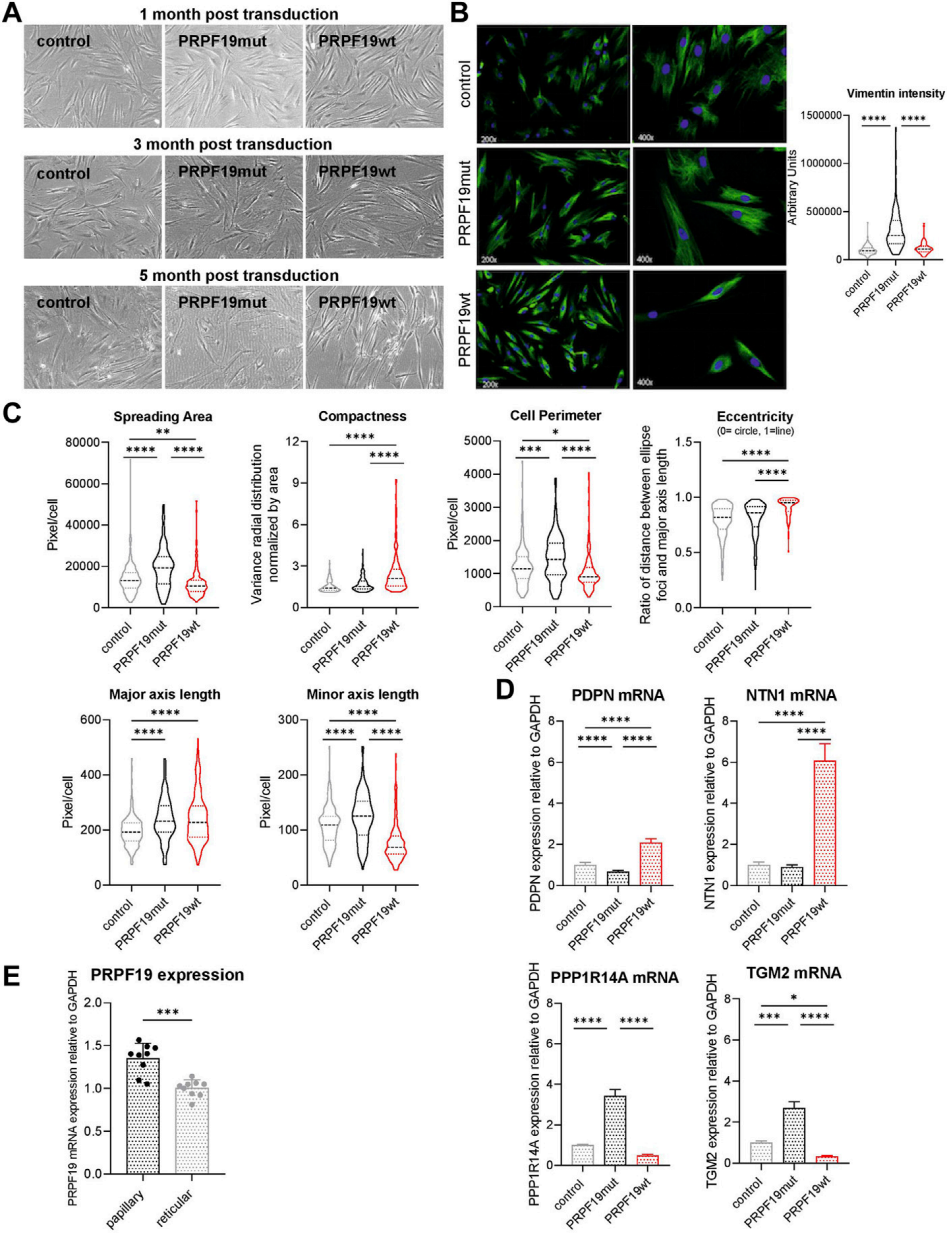
PRPF19 induces a papillary/juvenile morphology in human dermal fibroblasts. Fibroblast strains were cultivated for more than 5 months, and **(A)** representative bright field images were taken. For the quantitative morphological analysis, **(B)** anti-vimentin immune fluorescent staining images were used to **(C)** automatically calculate morphological parameters of the cells using CellProfiler. Cellular morphology was reflected by spreading area, compactness, cell perimeter, eccentricity (0 means the cell is a perfect circle and 1 means it is a prefect line), and the length of the major and minor axis. Data are plotted as violin plots. Statistical significance was determined by one-way ANOVA with Tukey correction for multiple comparisons. Six to seven pictures were analyzed for each fibroblast strain. **(D)** qPCR analysis of cell lysates for the papillary marker genes PDPN and NTN1 (upper panel) and the reticular marker genes PPP1R14A and TGM2 (lower panel). Data are presented as expression level normalized to control and are plotted as means + SD. Statistical significance was determined by one-way ANOVA with Tukey correction for multiple comparisons. **(E)** PRPF19 mRNA expression of primary papillary and reticular fibroblasts isolated from human skin of three different donors. Data are presented as expression level normalized to reticular fibroblasts and are plotted as means + SD. Statistical significance was determined by paired two-tailed Student’s t-test and assumption of equal variance (n = 3). *p < 0.05; **p < 0.01; ***p < 0.001; ****p < 0.0001.

During cultivation, we observed significant morphological differences between the three established fibroblast types. Fibroblasts transduced with empty control vector or phosphorylation-insufficient PRPF19 (PRPF19mut) showed a more reticular, flat and spread-out morphology early after transduction and developed a characteristic morphological senescent phenotype at later stages. In contrast, PRPF19wt has a more papillary-like morphological phenotype, characterized by long, slim, spindle-like cells that grew denser and were less contact-inhibited throughout the whole culture period of 5 months (Figures 3A, B).

To quantify morphological phenotypes and phenotypical changes observed by light microscopy, we performed immunofluorescence staining against vimentin to assess the cellular shape and area of the fibroblasts (Figure 3B). We quantified spreading area, compactness, eccentricity, and the length of the major and minor axis of the three cell types to describe their morphological phenotype. Evaluation of these parameters confirmed a reticular morphology of control and PRPF19mut fibroblasts (Figure 3C). PRPF19wt cells, however, showed a papillary and juvenile silhouette (Figure 3C), despite an overall increase in senescence markers (Figure 2).

In order to corroborate the morphologic observation by expression of papillary or reticular marker genes, we performed qPCR for Netrin-1 (NTN1) and podoplanin (PDPN) as papillary markers, as well as tissue transglutaminase-2 (TGM-2) and protein phosphatase 1 regulatory inhibitor subunit 14A (PPP1R14A) as reticular marker genes (Lämmermann et al., 2018; Janson et al., 2012). Indeed, in PRPF19wt, the papillary marker genes were significantly more abundant than those in control and PRPF19mut (Figure 3D upper panel). On the other hand, expression of the reticular markers TGM-2 and PPP1R14A was higher in control and PRPF19mut cell strains (Figure 3D, lower panel). In line with these findings, PRPF19 mRNA expression was significantly lower in primary reticular fibroblasts than in site-matched papillary fibroblasts isolated from human skin samples (Figure 3E).

In summary, these findings indicate that only the overexpression of wild-type PRPF19, but not its phosphorylation incompetent form, induces a morphological change to a more papillary/juvenile fibroblast phenotype, which is preserved even in high passages despite a shortened replicative lifespan and an increase in senescence markers. SNEVmut cells, however, show an even more reticular phenotype than control cells, underlining the importance of PRPF19 phosphorylation for the morphological phenotype in human skin fibroblasts.

### 3.4 PRPF19 overexpressing fibroblasts have an SASP-resembling activity despite a papillary-like phenotype

**FIGURE 4:**
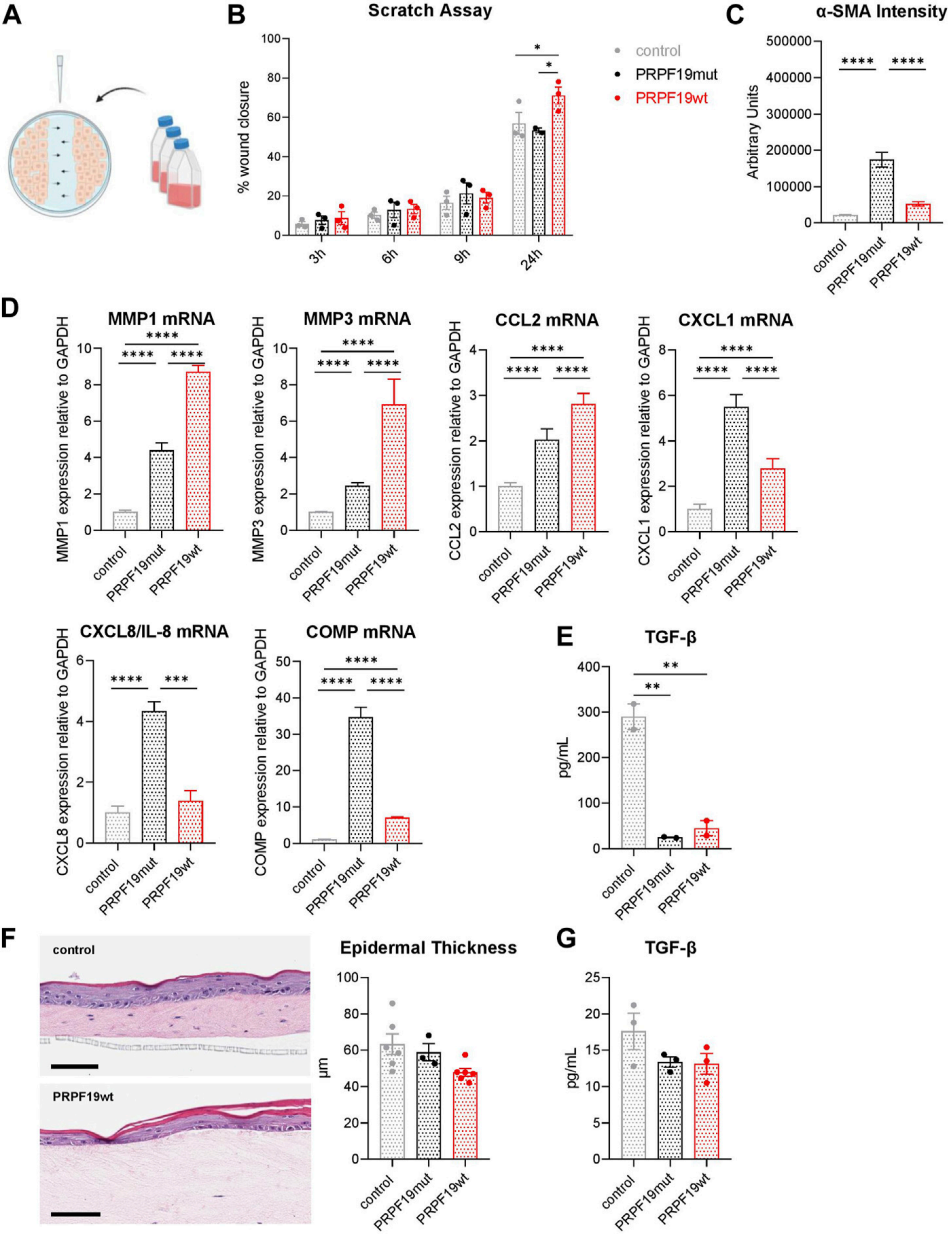
PRPF19 overexpressing fibroblasts have SASP-resembling activity despite a papillary-like phenotype. **(A)** Schematic representation of the experimental design for the scratch assays. When NHEKs reached confluence, a scratch was made throughout across the longitudinal axis of a 6-well tissue culture plate using a pipette tip. Scratched cell monolayers were cultivated with conditioned media of the three fibroblast strains, and wound closure was observed over 24 h. **(B)** Wound closure in % over a time course of 24 h. All data are plotted as means ± SEM of three independent experiments. Statistical significance was determined by two-way ANOVA with Tukey correction for multiple comparisons (n = 6). **(C)** Quantification of the signal intensity of *a*-SMA immune fluorescent staining. All data are plotted as means ± SEM. One-way ANOVA with Tukey correction for multiple comparisons. Duplicates were performed for each cell strain with an average of eight pictures analyzed for each replicate. **(D)** mRNA expression of genes involved in wound healing. Data are presented as expression levels normalized to control and are plotted as means +SD. Statistical significance was determined by one-way ANOVA with Tukey correction for multiple comparisons. **(E)** TGF-β1 ELISA of conditioned media from the three fibroblast strains (n = 2). Data are plotted as individual values ±SEM. Statistical significance was determined by one-way ANOVA with Tukey correction for multiple comparisons. **(F)** Full-thickness human skin equivalents were constructed as described in the Methods section. The average epidermal thickness was calculated by the height of the originated frame of the epidermis using ImageJ. Scale bar 100 µm (n = 3–6). Data are plotted as individual values ±SEM. Statistical significance was determined by one-way ANOVA with Tukey correction for multiple comparisons. **(G)** TGF-β1 ELISA of full-thickness skin equivalents generated with the three different fibroblast cells (n = 3). Data are plotted as individual values ± SEM. Statistical significance was determined by one-way ANOVA with Tukey correction for multiple comparisons. *p < 0.05; **p < 0.01; ***p < 0.001; ****p < 0.0001.

Wounding induces a high degree of heterogeneity among fibroblasts, and especially papillary fibroblasts are important for hair follicle formation in scarless wound healing (Driskell et al., 2013; Guerrero-Juarez et al., 2019; Rippa et al., 2019) and epidermal regeneration (El-Ghalbzouri et al., 2002). Since we observed a more papillary phenotype of PRPF19wt fibroblasts, we tested if the overexpression of PRPF19 in fibroblasts would also result in paracrine support of wound healing using keratinocyte scratch assays. Confluent keratinocyte monolayers were wounded and subsequently cultured in conditioned media from either the control, PRPF19wt, or PRPF19mut fibroblast cell types. Keratinocyte proliferation/migration into the wounded area was observed over 24 h (Figure 4A). Keratinocytes cultivated with conditioned media from PRPF19wt fibroblasts proliferated stronger/migrated faster into the wounded area than those cultured in conditioned media from PRPF19mut and control fibroblasts (Figure 4B, Supplementary Figure S2). This observation was most pronounced at 24 h post wounding. The slowest wound closure was observed for keratinocytes cultivated with media from PRPF19mut fibroblasts (Figure 4B). Thus, we conclude that PRPF19 overexpression in fibroblasts enhances keratinocyte proliferation/migration into wounded areas, and this is dependent on its phosphorylation ability at S149.

Wound healing capacity is also associated with contractility. We therefore, assessed a possible effect of PRPF19 on wound contraction by quantification of *a*-smooth muscle actin (α-SMA), a protein present in skeleton muscles belonging to the actin family and involved in the contractile apparatus, cell mobility, and wound contraction. *a*-SMA expression was higher in PRPF19wt than in control cells (Figure 4C). Since this indicated a beneficial role of PRPF19 in wound closure, we examined the expression of other genes with known roles in this process. Indeed, expression of matrix metalloproteinase (MMP) 1 and MMP3 was upregulated in PRPF19wt, indicating stronger ability to remodel the extracellular matrix in wounds (Figure 4D). Additionally, other genes involved in keratinocyte proliferation and wound healing, like C-C motif chemokine ligand (CCL) 2, C-X-C motif chemokine ligand (CXCL) 1, and CXLC8/IL-8, are upregulated in PRPF19wt compared with control (Figure 4D). Cartilage oligomeric matrix protein (COMP), recently shown to negatively influence keratinocyte proliferation and hypothesized to play a role in chronic non-healing wounds (Bozó et al., 2020), was expressed highest in PRPF19mut, which also showed delayed wound healing (Figures 4B,D). As transforming growth factor beta (TGF-β) plays an important role in regulating responses to skin injury, we had a closer look at the secreted TGF-β1 levels in the conditioned media of the transduced fibroblast cell types. Interestingly, we found that there was less TGF-β1 secretion of PRPF19wt as well as PRPF19mut compared to control fibroblasts (Figure 4E). To further evaluate the effect of PRPF19 overexpressing fibroblasts on skin development and homeostasis, we generated full-thickness skin equivalents (SE) with the different fibroblast cell types and investigated epidermal differentiation potential. There is a trend of epidermal thickness being decreased in PRPF19wt compared to control skin equivalents as well as the secreted TGF-β1 level; however, it is not as pronounced as before in monolayer culture conditions (Figure 4F).

Taken together, this indicates that PRPF19 overexpression in fibroblasts might enhance wound healing in cells autonomously, but not by paracrine effects on epidermal differentiation, and this process seems to be at least partly dependent on PRPF19 phosphorylation.

Collectively, PRPF19 overexpression induces shortened lifespan and expression of senescence markers (SA-β-gal, p21, and p53) and factors of the senescence-associated secretory phenotype (SASP) (Coppé et al., 2010), but at the same time, it induces better DNA damage repair and decreased TGFβ secretion independent of ATM phosphorylation. The overexpression of ATM phosphorylation-competent PRPF19 (PRPF19wt) induces a papillary phenotype (morphology and marker genes) and enhances keratinocyte migration/proliferation, but negatively affects keratinocyte differentiation in SE. Thus, by modulation of PRPF19 levels, the senescent phenotype is independently modified from cell morphology and support of wound healing capacity, which seem to be under the control of ATM-induced phosphorylation.

In this study, we showed that PRPF19 does not prolong the lifespan of human dermal fibroblasts but has a strong influence on their morphology and phenotype. PRPF19 overexpressing fibroblasts show a more papillary and juvenile morphology throughout their lifespan compared to control cells. Their transcriptional phenotype is highly heterogeneous, exhibiting papillary and reticular features. In addition, the secretome of these fibroblasts has a positive effect on wound healing, supporting keratinocyte repopulation of the wounded areas.

## 4 Discussion

PRPF19 is a ubiquitously expressed, essential (Fortschegger et al., 2007) ubiquitin E3 ligase (de Moura et al., 2018; Hatakeyama and Nakayama, 2003a) involved in regulating cellular functionalities like DNA repair, mRNA splicing, adipogenesis, and lifespan extension in human endothelial cells (Voglauer et al., 2006a) and in *D. melanogaster* (Garschall et al., 2017) and therefore, of fundamental importance for cellular survival. PRPF19 is one of the conserved PRP19-associated proteins and together with cell division cycle 5 like (CDC5L), pleiotropic regulatory locus 1 (PRL1), and pre-mRNA splicing factor (SPF27) forms the NineTeen Complex (NTC) or Prp19 complex (Prp19C) (Chanarat and Sträßer, 2013). Its involvement in regulating cellular senescence was recently confirmed by its knock-down that induces a senescence-like phenotype in human fibroblasts through the p53–p21 pathway (Yano et al., 2021). Here, we aimed to generate stable PRPF19 and phosphorylation-incompetent PRPF19 overexpressing human fibroblasts to test its functionalities in the context of *in vitro* skin physiology and aging.

Surprisingly, neither overexpression of PRPF19wt nor of PRPF19mut resulted in extension of cellular lifespan despite decreased levels of DNA damage markers with and without cisplatin treatment, but instead led to an accelerated entry into senescence in contrast to human endothelial cells, where we observed extension of cellular lifespan by both mutant and wild-type PRPF19 (Voglauer et al., 2006a). A cell type-dependent context of PRPF19 action on cellular senescence was also recently observed in liver carcinoma cells, where its overexpression as a myc-tag fusion protein resulted in induction of senescence (Yin et al., 2022), similarly as in our fibroblast experiments. Initial findings of PRPF19 levels and survival of cancer patients, where we observed higher survival correlating with high levels of PRPF19 (Voglauer et al., 2006a), which we interpreted at the time as a result of higher DNA repair capacity, now might also involve elevated induction of senescence as an additional tumor-suppressor mechanism.

Another observation reinforcing this hypothesis is that PRPF19wt fibroblasts, although showing all signs of senescence, morphologically maintain a papillary phenotype with higher DNA repair capacity, as shown by lower γH_2_AX levels, compared to control cells. Skin aging is associated with changes in proteoglycans and morphological changes in the papillary dermis as well as a decrease in the number of papillary fibroblasts due to their transition into reticular fibroblasts (Janson et al., 2013b; Sorrell and Caplan, 2004b). Reticular fibroblasts are described as flat and stellate (Mine et al., 2008), reminiscent of senescent cells. In contrast, a characteristic of papillary fibroblasts is the spindle-shaped morphology (Mine et al., 2008), which was observed after wild-type PRPF19 overexpression. This phenotype did not change even after prolonged culture of up to 5 months, indicating that PRPF19 might slow down the transition from papillary into reticular fibroblasts. This finding was supported by qPCR of NTN1 and PDPN, which served as papillary marker genes and were highly expressed in PRPF19wt compared to control fibroblasts, while reticular markers PPP1R14A and TGM-2 were lowly expressed. Interestingly, when looking at PRPF19 mRNA levels of the site-matched reticular and papillary fibroblasts, we observed that PRPF19 is significantly higher in the papillary versus the reticular subpopulation. We, therefore, assume that PRPF19 changes the proportional distribution of papillary and reticular fibroblasts, either by decelerating the differentiation of papillary to reticular fibroblasts or by promoting the expression of papillary marker genes. Future investigations are necessary to determine the exact mechanism, but a role of PRPF19 in various cell differentiation settings has already been observed (Urano et al., 2006; Khan et al., 2017; Allen et al., 2022). PRPF19mut, which cannot be phosphorylated by ATM at the targeted serine, showed increased senescence and showed a clear reticular phenotype in contrast to PRPF19wt, indicating that the phosphorylation of PRPF19 might be necessary to modulate the morphology of fibroblasts.

Papillary and reticular fibroblasts have a distinct influence on keratinocyte proliferation and behavior (Sorrell and Caplan, 2004b; Janson et al., 2017; Janson et al., 2012), which could be further highlighted by our approach to study wound healing and keratinocyte differentiation. Scratch assays displayed the beneficial influence of the secretome of PRPF19wt fibroblasts, which accelerated the repopulation of wounded areas with keratinocytes. This can be interpreted in two different ways: either the more papillary-like phenotype of PRPF19wt fibroblasts, reported to be similar to upper wound fibroblasts, which are important for scarless wound healing and epidermal regeneration (El-Ghalbzouri et al., 2002; Driskell et al., 2013; Guerrero-Juarez et al., 2019; Rippa et al., 2019), is responsible or, as an alternative explanation, the SASP that we recently observed to promote scratch closure (Terlecki-Zaniewicz et al., 2019), is responsible. Interestingly, phosphorylation of PRPF19 is essential as keratinocytes cultivated with conditioned media from PRPF19mut fibroblasts display poorest wound closure. Upregulation of the SASP factors (Coppé et al., 2008; Gorgoulis et al., 2019), MMP1 and MMP3, which are important for matrix remodeling, as well as elevated levels of CXCL1 and IL-8, which both promote keratinocyte proliferation, migration, and reepithelialization (Rennekampff et al., 1997; Rennekampff et al., 2000) further support the hypothesis that PRPF19 might influence and indirectly accelerate wound healing. One possible key regulatory element in this context might be the TGF-β pathway. TGF-β is a molecule extensively studied in the context of wound healing, and its effect on keratinocyte reepithelialisation seems paradoxical. After wounding, keratinocytes upregulate TGF-β1 expression, a factor known to suppress their proliferation and differentiation (Coffey et al., 1988; Sellheyer et al., 1993). However, there are several studies showing that TGF-β1 is not essential for reepithelialization, but quite the opposite, that is, neutralizing antibodies as well as knock-out models exhibited even accelerated wound reepithelialization (Amendt et al., 2002; Werner and Grose, 2003; Koch et al., 2000). These observations support our hypothesis that PRPF19 overexpressing fibroblasts might enhance early re-establishment of the epithelial barrier by reduced TGF-β1 production, resulting in enhanced keratinocyte proliferation and migration to the early wound beds. Garlick et al. showed the same phenomenon in a 3D wound setting but could, in addition, demonstrate that the negative effect of TGF-β1 on keratinocyte proliferation and wound reepithelialization is dose-dependent and reduced over time (Garlick and Taichman, 1994). There are also several reports indicating a positive effect of TGF-β1 on wound healing. It was shown to regulate MMPs (Madlener et al., 1998; Ellenrieder et al., 2001; Verrecchia et al., 2001) and integrins (Gailit et al., 1994; Zambruno et al., 1995) important for keratinocyte and fibroblast migration, stimulate wound contraction (Montesano and Orci, 1988), and accelerate wound healing in several experimental animal models (Salomon et al., 1990; Beck et al., 1991; Beck et al., 1993). Nevertheless, clinical trials of TGF-β1 administration on chronic ulcers have only achieved little effect (Komeliagina and Antsiferov, 2019; Mulder, 2004). This highlights the need for further studies investigating the effects of TGF-β1 in all stages of wound healing, especially in *in vivo* setting. Further research is also necessary to investigate the connection between PRPF19 and TGF-β and its versatile role in skin homeostasis and wound healing.

## 5 Conclusion

With this work, we could gain insights into the complex, multitalented human protein PRPF19 and its ability to influence diverse molecular mechanisms in human dermal fibroblasts. Especially its capability to influence fibroblast phenotypes and functions and the resulting positive effect on epidermal wound healing was nicely shown. Further research is necessary to investigate how PRPF19 is exactly involved in these pathways. Given the fact that overexpression of PRPF19 in fibroblasts resulted in a different phenotype than in endothelial cells, its effect seems to be highly dependent on the cell line transduced, and it would be of significant interest to further investigate its influence on other cell types.

## Data Availability

The original contributions presented in the study are included in the article/Supplementary Material; further inquiries can be directed to the corresponding author.
