# Supplementary figures and images for: PRPF19 modulates morphology and growth behavior in a cell culture model of human skin
Source: Front Aging. 2023 May 5;4:1154005. doi: 10.3389/fragi.2023.1154005 (PMC10196211; doi:10.3389/fragi.2023.1154005)

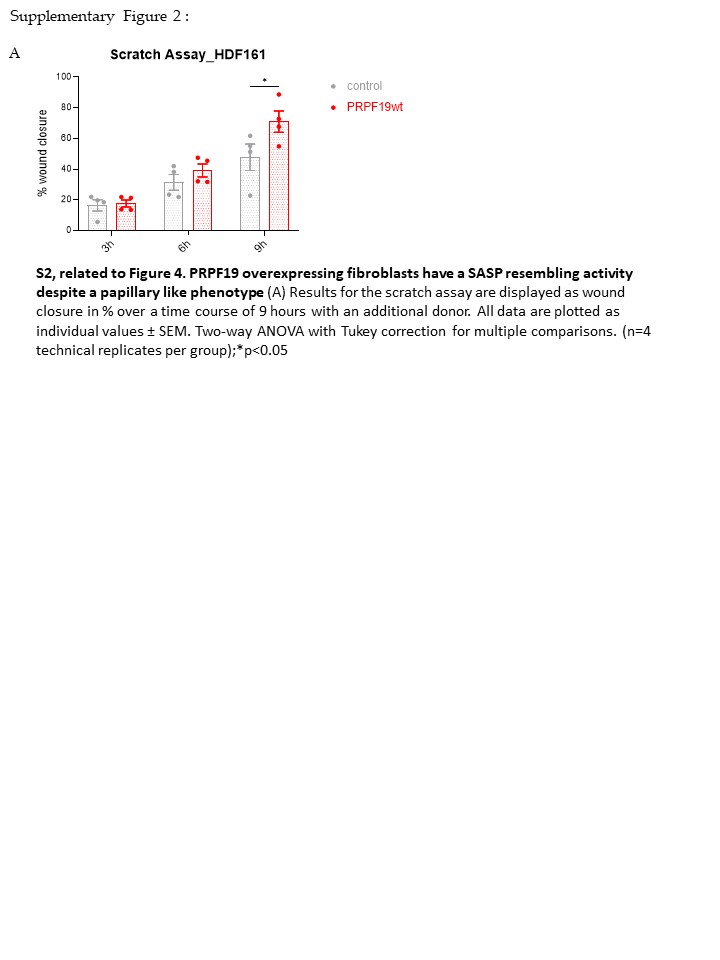

Supplement: Supplementary file 1 [file Image3.JPEG]

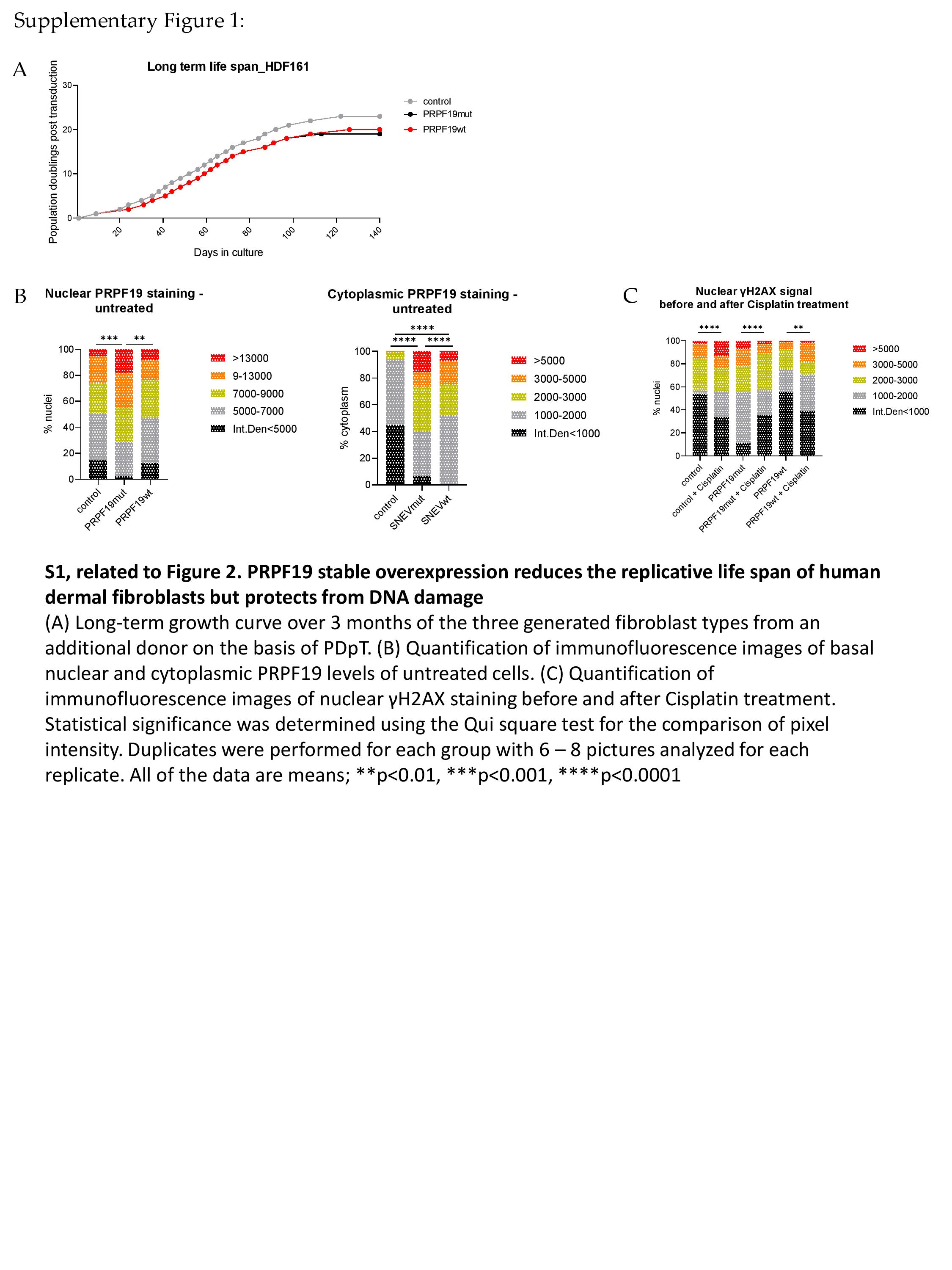

Supplement: Supplementary file 2 [file Image2.jpg]

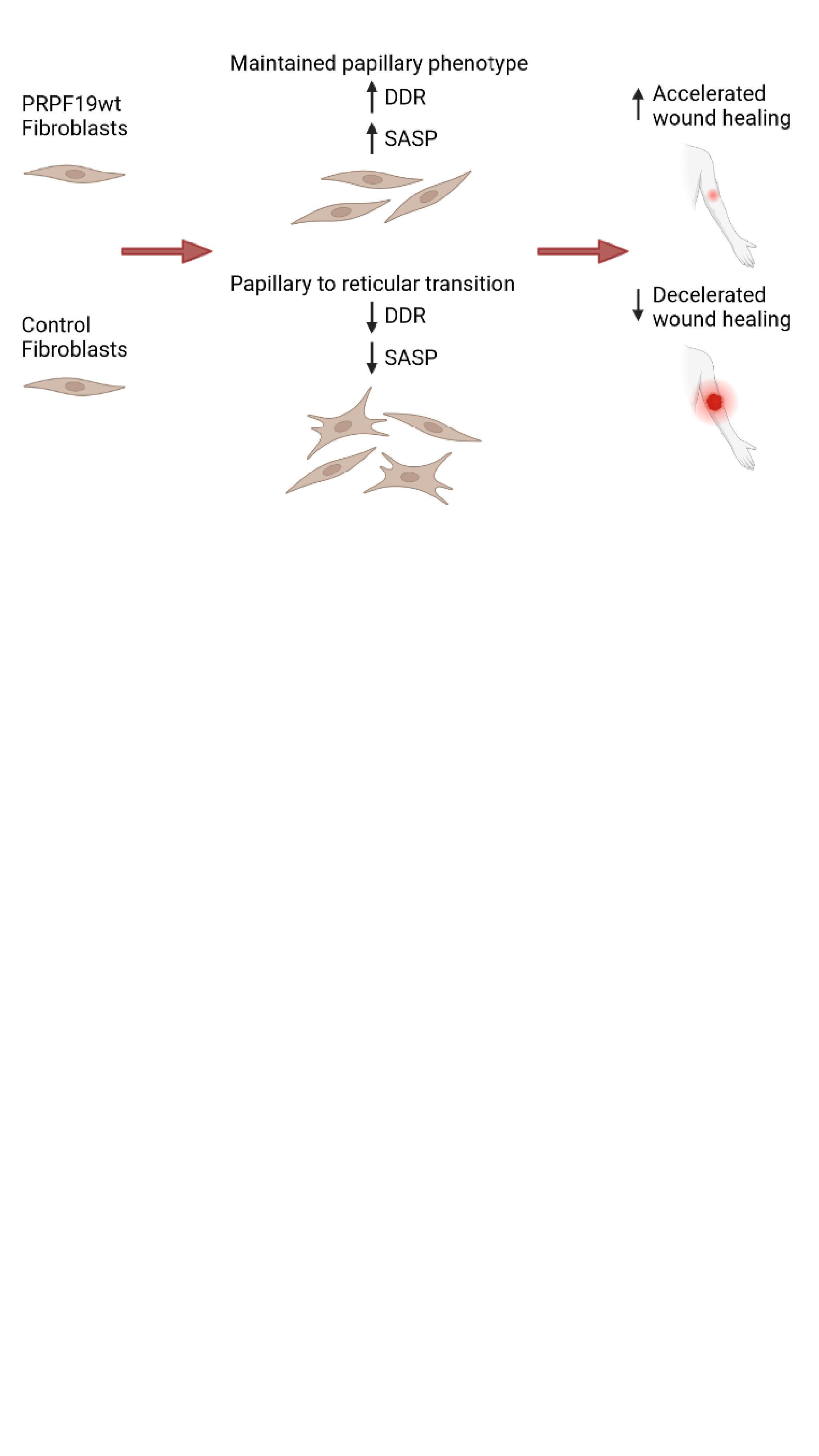

Supplement: Supplementary file 3 [file Image1.JPEG]
